# CT-derived density of intracranial arteriosclerosis: a population-based cohort study

**DOI:** 10.1007/s00330-025-12180-1

**Published:** 2026-01-15

**Authors:** Bernhard P. Berghout, Robin Y. R. Camarasa, Maarten J. G. Leening, Marleen de Bruijne, M. Kamran Ikram, Daniel Bos

**Affiliations:** 1 Department of Epidemiology, Erasmus MC–University Medical Center, Rotterdam, The Netherlands; 2 Department of Radiology and Nuclear Medicine, Erasmus MC–University Medical Center, Rotterdam, The Netherlands; 3 Cardiovascular Institute, Department of Cardiology, Erasmus MC–University Medical Center Rotterdam, Rotterdam, The Netherlands; 4 Department of Computer Science, University of Copenhagen, Copenhagen, Denmark; 5 Department of Neurology, Erasmus MC–University Medical Center, Rotterdam, The Netherlands

**Keywords:** Arteriosclerosis, Multidetector computed tomography, Cerebral arterial diseases, Cerebrovascular disorders

## Abstract

**Background:**

The CT-derived density of coronary artery calcification is increasingly associated with the risk of ischemic heart disease. Whether this principle also applies to intracranial artery calcifications (IAC) and cerebrovascular disease risk is unknown, primarily due to the lack of population-based estimates of IAC density and its determinants. We investigated these facets in this cohort study.

**Materials and methods:**

In 2464 community-living individuals who underwent non-contrast CT, we measured IAC density and assessed its correlation with IAC volume using Spearman’s ρ. We described its distribution in intracranial carotid artery calcification (ICAC), with specific estimates for its subtypes, and vertebrobasilar artery calcification (VBAC). We investigated associations between risk factors and IAC density using multivariable ordinal regression models.

**Results:**

The prevalence of IAC was 82.8%, with a median density of 232 (IQR 189–287) HU. IAC density correlated moderately with volume (ρ 0.67, 95% CI [0.65–0.70]). ICAC was predominantly composed of higher density, with 80.1% of affected participants having components of ICAC above 400 HU, whereas only 32.0% of participants with VBAC had components above 400 HU. Intimal subtype ICACs showed a predominance for lower densities when compared to medial subtype ICACs. The main determinants of IAC density were hypertension, use of lipid-lowering medication, and smoking, with adjusted odds ratios of 1.59 [1.28–1.90], 1.55 [1.26–1.91], and 1.33 [1.10–1.61], respectively.

**Conclusion:**

IAC density differs significantly between the anterior and posterior cerebropetal arteries. While IAC density correlated only moderately with its volume, the associations between cardiovascular risk factors and IAC density were mostly similar to those observed with IAC volume.

**Key Points:**

***Question*** *Drivers of the CT density of intracranial artery calcifications are unknown and may reveal novel risk targets for population-based prevention strategies.*

***Findings*** *Calcifications of the anterior cerebral circulation are denser than those of the posterior circulation. Hypertension, diabetes, and smoking are key drivers of calcification density, resembling most drivers of its volume.*

***Clinical relevance*** *Calcification density may serve in distinguishing subtypes of intracranial calcifications, improving detection of subtype-specific effects. Further research is warranted to determine the role of intracranial arteriosclerosis density in prevention strategies for cerebrovascular diseases.*

**Graphical Abstract:**

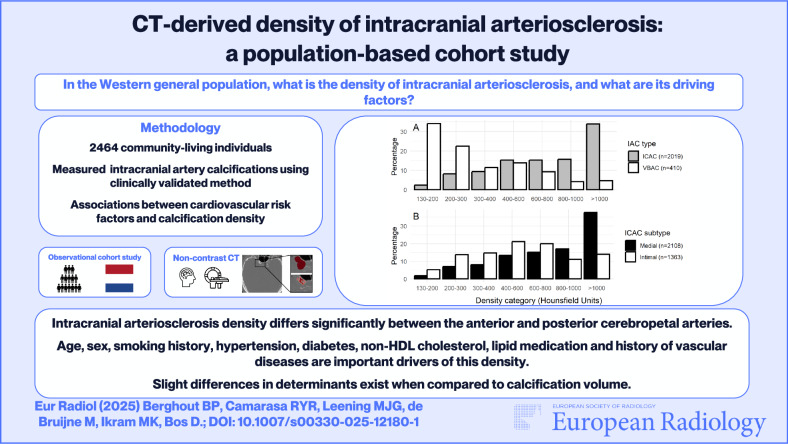

## Introduction

Intracranial artery calcification (IAC), the defining feature of intracranial arteriosclerosis on CT imaging [1], is a recognized risk factor for both stroke [2–4] and dementia [5]. To date, research has concentrated on exploring the causes and consequences of the prevalence and volume of IAC [1]. Insights from research in the field of coronary artery calcification (CAC) indicate that the density of calcification may have additional informative value over calcification volume, as higher density reflects more stable atherosclerotic disease [6, 7]. However, it is unknown whether this association extends to IAC due to several specific knowledge gaps in the literature.

First, current literature on IAC density is sparse and limited to descriptions from patient studies using contrast-enhanced imaging, which precludes the detection of low-density calcifications [8]. Second, the determinants of IAC density are unknown, making it unclear whether cardiovascular risk factors influence the density of IAC. Finally, density is an overlooked subject of potential relevance to the defining characteristic of IAC, which is the presence of two distinct morphological patterns of calcification: calcification of the internal elastic lamina (medial subtype), versus calcification of the intimal layer (intimal subtype). Calcifications in the medial subtype affect extensive lengths of intracranial arteries, while intimal calcifications are typically associated with localized atherosclerotic disease. Distinct differences in determinants and implications for cerebrovascular health exist between these subtypes. Medial calcifications are associated with diabetes, vascular diseases [2, 9], and increased incidence of white matter hyperintensities [10], dementia [5], worse functional outcome following intravenous thrombolysis [11], and better functional outcome following endovascular therapy in ischemic stroke [12]. In contrast, intimal calcifications are associated with smoking, hypertension [2, 9], and better collateral status on CT imaging [13]. Whether density also differs between these distinct subtypes is unknown, yet this seems plausible given their differing determinants, and could aid in a more accurate distinction between the two subtypes. To determine whether calcification density plays a similar role in intracranial arteriosclerosis as it does in coronary artery calcification, a thorough investigation of its magnitude, distribution, and determinants is first required. Therefore, we described the density of IAC and its determinants in the general population.

## Materials and methods

### Setting

This study is embedded in the Rotterdam Study, a population-based, prospective observational cohort study among older Dutch adults [14]. The Rotterdam Study was initiated in 1990 with 7983 participants and expanded in 2000 with 3011 participants, all aged ≥ 55 years at participation.

### Study population and image acquisition

**Fig. 1:**
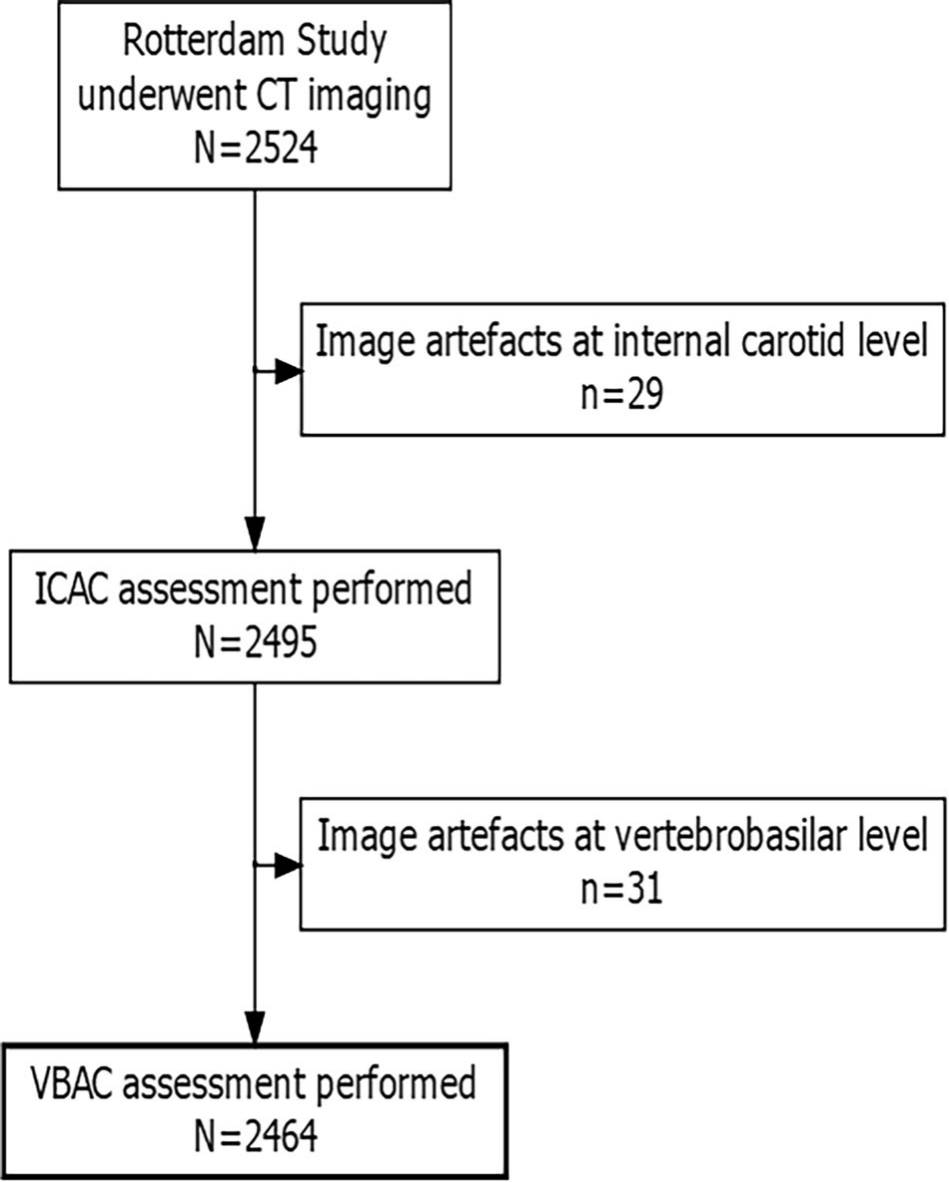
Flowchart depicting the selection process of the study population. ICAC, intracranial carotid artery calcification; VBAC, vertebrobasilar artery calcification; N, total count remaining; n, count excluded

Participants attending follow-up visits between 2003 and 2006 at the research center were invited to undergo non-contrast-enhanced MDCT imaging of the intracranial arteries, as part of a CT imaging protocol measuring arterial calcification throughout the body [15]. A total of 2524 participants were scanned. However, due to image artefacts obscuring the view of the intracranial arteries, 60 examinations were not gradable. This left a total of 2464 participants available for analysis (Fig. 1). A 16-slice (*n* = 773) or 64-slice (*n* = 1691) MDCT scanner (Somatom Sensation 16 or 64, Siemens) was used for non-contrast MDCT imaging. The imaging field-of-view and settings were optimized for the evaluation of the intracranial arteries [16]. Detailed information regarding imaging parameters is described elsewhere [17].

### Assessment of demographic and cardiovascular determinants

At the study entrance, all participants were invited for blood sampling and physical examination at the research center. Blood pressure was measured in a seated position after a resting period of 5 min on the right upper arm using a random-zero sphygmomanometer. We defined hypertension as either a systolic blood pressure ≥ 140 mmHg, a diastolic blood pressure ≥ 90 mmHg, or the use of blood pressure-lowering medication. Diabetes mellitus was defined as having either a serum non-fasting glucose level ≥ 11.1 mmol/L, a fasted glucose level ≥ 7.0 mmol/L or the use of glucose-lowering medication. Data on the history of coronary heart disease (CHD) and cerebrovascular accidents (CVA), including stroke and transient ischemic attack (TIA), were collected in the following manner. A baseline home interview is conducted after entrance to the Rotterdam Study, where trained nonmedical interviewers administer a standardized questionnaire to participants to obtain information on their medical history, health status (symptom history, e.g., (hemi-)paresis, chest discomfort, breathlessness, etc.), labels of their current medication use and their indication, and smoking habit. This information is cross-referenced with information on prevalent disease status obtained from the Nationwide Medical Registry (Landelijke Medische Registratie, replaced by the Landelijke Basisregistratie Ziekenhuiszorg on the 1st of January 2014, governed by the Dutch Ministry of Healthcare in Utrecht, The Netherlands). In addition to this registry, the study database is automatically linked with participants’ files from their general practitioners’ offices, where their general practitioner acts as a gatekeeper for access to further specialized care and as the coordinator of each individual’s medical information. Clinical information from these general practitioner offices is obtained for each participant and, after a meticulous process where medical documents are screened by hand, used in combination with the aforementioned sources (interview, Nationwide Medical Registry, general practitioner database) to obtain a complete overview of each participant’s health status at baseline [18, 19]. Any coronary revascularization, myocardial infarction, or CVA occurring before the date of imaging was classified as prevalent disease.

### Measures of intracranial arteriosclerosis

The assessment of IAC was performed using an established methodological framework that is frequently applied in intracranial arteriosclerosis research [8, 9, 20–22], which has previously been clinically validated [23, 24] and assessed for its inter-rater reliability. The intraclass correlation coefficient of this method was 0.99 based on ratings of two observers on 50 CT examinations [25], which was also found in a different study where the coefficient of variation for the interobserver differences was 0.07 [26]. An elaborate description of this methodology is available in the Supplementary Material A.

As an extension to this framework, the assessment of average arterial calcification density was performed using a custom-made, Python-based script (available from https://gitlab.com/radiology/aim/carotid-artery-image-analysis/kalk), as described previously [8]. For each artery, average densities were automatically calculated by summing the HU values of voxels with an HU ≥ 130 within the region of interest and dividing this sum by the number of these voxels. Average IAC, ICAC and VBAC densities were subsequently derived by summing average HUs per artery and dividing by the number of arteries affected by arterial calcification.

**Fig. 2:**
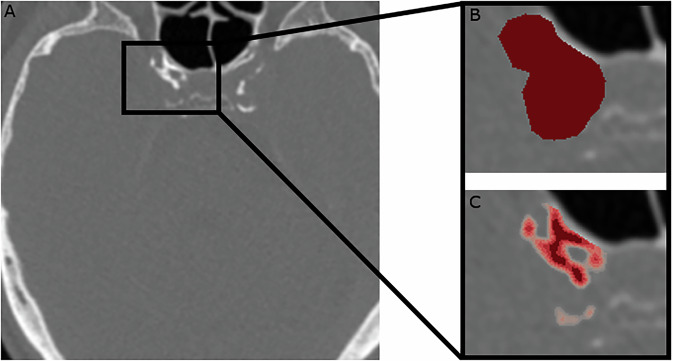
Axial slice examples of right intracranial carotid artery calcification. The middle of the region of interest is depicted in **A**. The manual delineation of the same slice is shown in **B**, while the corresponding components of increasing HU density are illustrated in intensifying shades of red in **C**

As CT-based density is suggested to reflect the maturity of arterial calcification [27], we repeated the volume and density measurements after changing the HU threshold to 200, 300, 400, 600, 800, and 1000 HU, in order to assess different possible stages of arterial calcification. An example of the different density components that are identified through this method is illustrated in Fig. 2. This enabled us to form a categorization of the highest density components measured in the arterial calcifications.

### Statistical analysis

Baseline characteristics of the study population are presented in means and standard deviations (SD) for normally distributed continuous variables, medians and interquartile range (IQR) for skewed continuous variables, or frequencies and percentages for categorical variables. Data on covariates were missing in 5.1% of participants, most frequently due to missing information on smoking behavior in 2.6% of participants. The mice algorithm was used to impute missing data, using five iterations and five imputations.

We first present the distribution of the highest category of density of IAC, categorized into seven groups (130–200, 200–300, 300–400, 400–600, 600–800, 800–1000, and > 1000). This is visualized through bar plots stratified by IAC type, with each bar representing the percentage composition of the highest category of density measured for participants with ICAC and participants with VBAC. Subsequently, we examined the variation in this distribution among ICAC subtypes at the artery level. In this analysis, each bar represents the percentage of the highest category of density measured among all internal carotid arteries, stratified by either medial or intimal calcification subtype. As we noticed a considerable difference in the distribution of the highest density category between the ICAC subtypes, we investigated whether density could aid in distinguishing the ICAC subtypes. To that end, we stratified arteries affected by ICAC based on the full morphological subtype score [20], introducing the “ambiguous” category with scores of five through eight representing those ICACs where there may be considerable disagreement between observers in the choice of the underlying subtype, and created boxplots displaying the distribution of mean density per stratum. Pairwise two-sided Wilcoxon tests were used to compare these distributions across the strata.

Next, we estimated Spearman correlation coefficients between IAC volume and IAC mean density to assess whether there was a monotonic association between these entities, and illustrate this association using a scatterplot. As these continuous measurements of IAC were both right-skewed, we applied a natural log-transformation to them to ensure a normal distribution prior to any statistical analyses. Next, in participants with IAC, we then assessed determinants of IAC density using a multivariable ordinal regression model on the highest category of density seen, incorporating the variables age, sex, scanner type (16-slice versus 64-slice), smoking behavior with never smoking as a reference, obesity, hypertension, diabetes mellitus, serum non-high-density lipid (HDL) cholesterol, use of lipid-lowering medication, prevalent CHD and CVA.

For sensitivity analyses, we first assessed determinants of IAC density in participants with IAC but free from any prevalent CHD or CVA, or the use of lipid-lowering medications. Lastly, we compared these determinants of IAC density with IAC volume in participants with IAC, using a linear regression model on the natural log-transformed continuous measurements, with the same adjustment strategy as that of the ordinal regression model. Pooled results of regression analyses on imputed datasets are presented. Analyses were performed in R (version 4.2.2), with the packages RVAideMemoire (0.9.83.7), MASS (7.3.58.3), mice (3.16.0) and ggpubr (0.5.0).

## Results

### Participant characteristics

Characteristics of 2464 participants at imaging are summarized in Table 1. Participants were 69.7 (SD 6.8) years old on average, and 51.9% were female. The prevalence of IAC was 82.8%, driven predominantly by a high prevalence of ICAC. The median volume of IAC was 67 (IQR 22–189) mm³, and the median density of IAC was 232 (IQR 189–287) HU.

**Table 1:** Baseline characteristics of the study population

Characteristic	Mean/count (SD/%)
Total sample size	2464
Age at imaging, years	69.7 (6.8)
Female sex	1280 (51.9)
Caucasian ethnicity	2395 (97.2)
Body mass index, kg/m²	27.7 (4.0)
Obesity, > 30 kg/m²	593 (24.1)
Smoking behavior	
Never	682 (27.7)
Former	1341 (54.4)
Current	376 (15.3)
Hypertension	1828 (74.2)
Systolic blood pressure, mmHg	147.0 (20.3)
Diastolic blood pressure, mmHg	80.1 (10.8)
Blood pressure-lowering medication use	990 (40.2)
Diabetes mellitus	276 (11.2)
Total cholesterol	5.7 (1.0)
HDL cholesterol	1.4 (0.5)
Serum non-HDL cholesterol	4.2 (1.0)
Lipid-lowering medication use	600 (24.4)
History of coronary heart disease	227 (9.2)
History of cerebrovascular accident	154 (6.3)
ICAC	
Presence	2019 (81.9)
Arteries visually deemed to be affected	3383 (68.6)^a^
Morphological subtype per score	
Intimal, 0–4	692 (20.5)
Ambiguous, 5–8	1914 (56.6)
Medial, 9–19	777 (23.0)
Predominantly medial subtype	987 (40.1)
Predominantly intimal subtype	774 (31.4)
Predominantly mixed subtype	258 (10.5)
Volume, mm³	146 (202)
Average density, HU	253 (73)
VBAC	
Presence	410 (16.6)
Volume, mm³	40 (108)
Average density, HU	188 (57)

*kg* kilogram, *m* meter, *HDL* high-density lipoprotein, *ICAC* intracranial carotid artery calcification, *VBAC* vertebrobasilar artery calcification, *mm* milimeter, *HU* Hounsfield unit

^a^ Percentage of all internal carotid arteries, i.e., 2464 participants * 2 arteries; percentages of morphological score categories correspond to the number of internal carotid arteries visually identified as having ICAC. Data was deemed missing for the variables: blood pressure (0.7%), BMI (0.4%), medication use (1.5%), serum markers (1.6%), smoking (2.6%)

### Distribution of density in intracranial arteriosclerosis

**Fig. 3:**
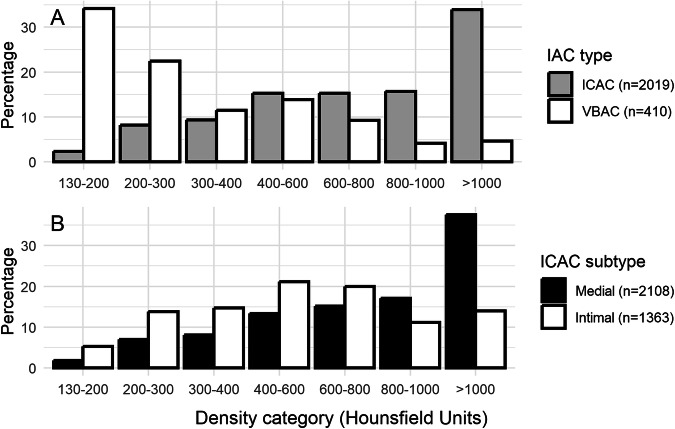
Distributions of maximum density detected among participants with IAC. Each bar indicates the percentage of each density category per total prevalence. In **A**, these percentages are illustrated separately per IAC subtype. In **B**, the percentages reflect maximum density category found among all arteries affected by ICAC, stratified for either medial or intimal subtype separately. IAC, intracranial artery calcification; ICAC, intracranial carotid artery calcification; VBAC, vertebrobasilar artery calcification; n, number

The distribution of the highest category of density measured in the 2040 patients with detectable IAC is illustrated in two panels of Fig. 3. In Fig. 3A, percentage distributions stratified by anterior or posterior cerebral circulation are shown. An increase in the percentage distribution across the highest density categories is observed for ICAC, whereas a decrease is noted for VBAC. Similarly, the trends in percentage distribution for ICAC subtypes are seen in Fig. 3B. The intimal subtype of ICAC predominantly exhibits lower maximum densities, whereas higher maximum densities for the medial subtype are observed.

**Fig. 4:**
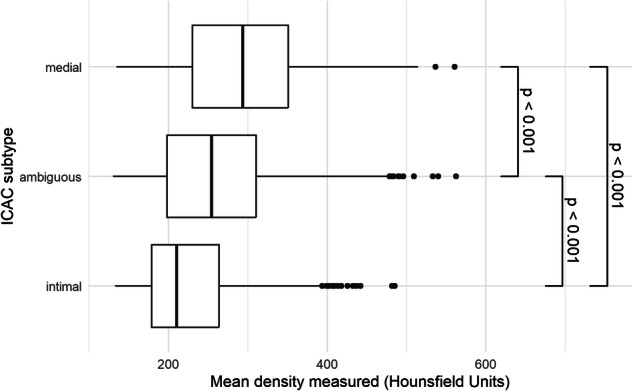
Stratified boxplots depicting the distribution of density per ICAC subtype. These boxplots depict the mean density among 3383 arteries visually identified to have ICAC, stratified by category of Kockelkoren score, with a score of < 5 reflecting intima type, 5–8 reflecting the ambiguous type and > 8 the medial type. ICAC, intracranial carotid artery calcification; CT, computed tomography. *p*-values refer to the results of two-sided *t*-tests

The densities for arteries affected by intimal calcification had a median HU of 210 (IQR 179–264), this was 294 (230–351) HU for medial calcification, and 255 (198–310) HU for arteries with an ambiguous subtype. These ranges and results of comparison tests are illustrated in Fig. 4, which highlights significant differences in density across the subtypes. Specifically, medial calcifications exhibit higher densities when compared to intimal calcifications.

### Correlation between density and volume of intracranial arteriosclerosis

**Fig. 5:**
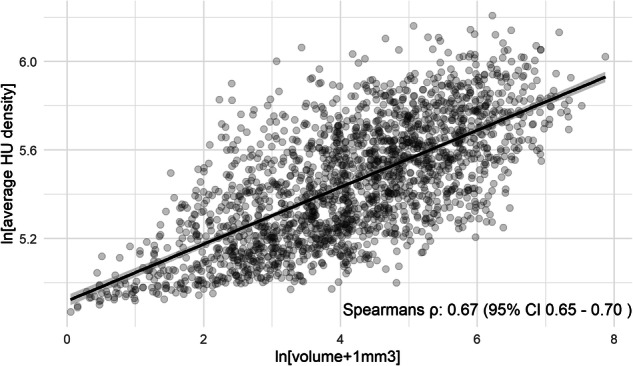
Scatterplot with Spearman’s ρ depicting correlation between IAC density and volume. ln, natural log; HU, Hounsfield unit; CI, confidence interval

There was moderate correlation between the mean density of IAC and the volume of IAC, with a Spearman’s ρ of 0.67 (95% confidence interval (CI) 0.65–0.70; Fig. 5), *p* < 0.01.

### Determinants of intracranial arteriosclerosis density

The results of multivariable ordinal logistic regression analysis on the maximum density measured in participants with IAC are displayed in Table 2. Participants who were older, on lipid-lowering medication, with cardiovascular risk factors other than obesity, or had a history of vascular disease showed a higher density of IAC on imaging. These findings remained similar after excluding 680 participants with a history of CHD or CVA, or who used lipid-lowering medication, although the association between diabetes mellitus and higher density was no longer statistically significant (Table 3).

**Table 2:** Results of multivariate ordinal logistic regression analyses in participants (*n* = 2040) with intracranial artery calcification

Characteristics	Adjusted^a^ common OR (95% CI)
Age in years, per year increase	1.07 (1.06–1.08)^b^
Female sex	0.65 (0.54–0.77)^b^
Ever smoking	1.35 (1.11–1.63)^b^
Obesity	1.16 (0.96–1.39)
Hypertension	1.59 (1.30–1.94)^b^
Diabetes	1.40 (1.09–1.79)^b^
Serum non-HDL cholesterol, per 1 mmol/L increase	1.14 (1.04–1.25)^b^
Use of lipid medication	1.58 (1.28–1.94)^b^
History of coronary heart disease	2.59 (1.89–3.54)^b^
History of cerebrovascular accident	1.56 (1.11–2.19)^b^
64-slice scanner used	0.94 (0.79–1.12)

Results depict the adjusted common OR for an increase in the category of the highest detected HU density component of intracranial artery calcification

*OR* odds ratio, *CI* confidence interval, *HDL* high-density lipoprotein

^a^ Adjustment model included all characteristics as covariates

^b^ *p* < 0.05

**Table 3:** Results of multivariate ordinal logistic regression analyses in *n* = 1360 participants with intracranial artery calcification and without a history of coronary heart disease, cerebrovascular accidents or the use of any lipid-lowering medication

Characteristics	Adjusted^a^ common OR (95% CI)
Age in years, per year increase	1.06 (1.05–1.08)^b^
Female sex	0.59 (0.48–0.73)^b^
Ever smoking	1.31 (1.05–1.63)^b^
Obesity	1.07 (0.85–1.35)
Hypertension	1.53 (1.23–1.90)^b^
Diabetes	1.21 (0.88–1.68)
Serum non-HDL cholesterol, per 1 mmol/L increase	1.21 (1.09–1.35)^b^
64-slice scanner used	0.87 (0.70–1.08)

Estimates show adjusted common OR and increase in the category of the highest detected HU density component of IAC

*HDL* high-density lipoprotein, *OR* odds ratio

^a^ Adjustment model included all characteristics as covariates

^b^ *p* < 0.05

Most effects seen in the ordinal logistic regression analysis were similar in the linear regression analysis of continuous increases in average IAC density. The only difference was found for those with a history of CVA, who no longer showed a statistically significant association with increasing density (Table 4). These results were generally similar in analyses of IAC volume, except that a higher serum non-HDL cholesterol and smoking no longer showed a statistically significant association with increasing IAC volume. Conversely, a history of CVA did show a statistically significant association with increasing IAC volume.

**Table 4:** Results of multivariate linear regression analyses on per SD increases in log-transformed IAC volume and average density, restricted to *n* = 2040 participants with IAC

	ß^a^ per SD increase (95% CI)	
Characteristics	Ln[Average density]	Ln[Volume + 1 mm³]
Age in years, per year increase	0.02 (0.01–0.03)^b^	0.05 (0.04–0.05)^b^
Female sex	−0.26 (−0.35 to −0.17)^b^	−0.15 (−0.24 to −0.07)^b^
Ever smoking	0.17 (0.07–0.27)^b^	0.06 (−0.03 to 0.16)
Obesity	0.03 (−0.07 to 0.13)	0.03 (−0.06 to 0.13)
Hypertension	0.21 (0.11–0.31)^b^	0.18 (0.08–0.28)^b^
Diabetes	0.18 (0.05–0.30)^b^	0.25 (0.13–0.37)^b^
Serum non-HDL cholesterol, per 1 mmol/L increase	0.08 (0.03–0.12)^b^	0.04 (−0.01 to 0.08)
Use of lipid medication	0.23 (0.12–0.33)^b^	0.20 (0.10–0.30)^b^
History of coronary heart disease	0.51 (0.36–0.65)^b^	0.40 (0.26–0.54)^b^
History of cerebrovascular accident	0.13 (−0.04 to 0.29)	0.22 (0.06–0.38)^b^
64-slice scanner used	−0.08 (−0.18 to 0.01)	−0.07 (−0.15 to 0.02)

*HDL* high-density lipoprotein

^a^ Adjustment model included all characteristics as covariates

^b^ *p* < 0.05

## Discussion

In this population-based cohort study among older adults, we report specific patterns of density in intracranial arteriosclerosis. Furthermore, IAC density correlated only moderately with IAC volume, implying that some differences exist between these two quantifiers of IAC. Additionally, specific differences in the risk factors of density and volume were observed. Hypertension, ever smoking and increasing serum non-HDL cholesterol were independently associated with increased density, while the latter two were not associated with volume. Finally, we demonstrated that density could act as another distinguishing factor in the identification of ICAC subtype, possibly facilitating more accurate establishment of ICAC subtype in ambiguous cases where the exact subtype is not obvious.

Previous estimates on the density of IAC are sparse, with the only other estimate from non-contrast CT images originating from a retrospective cross-sectional study of patients with acute ischemic stroke [28]. This study reported only maximum IAC density stratified by symptomatic side, complicating direct comparisons with the present study’s findings. Another study reporting IAC density estimates used CT-angiography images from patients with recent ischemic stroke and TIA to identify IAC and determine its mean density [8]. The contrast-enhanced images necessitated setting a higher HU threshold to distinguish arterial calcification from luminal contrast, which again complicates comparisons with findings from the present study, as this precludes the assessment of lower-density calcifications. However, this study also observed a similar pattern of higher ICAC density compared to lower VBAC density. We propose three possible explanations for this particular pattern. First, the vertebrobasilar arteries differ morphometrically from the intracranial carotid arteries in terms of length, caliber and tortuosity, and have greater anatomical variation [29]. Second, previous publications from this study suggest that while VBAC shares a similar pathophysiology to ICAC, it may start to develop later in life compared to ICAC [15, 30]. Third, approximately 70% of cerebrovascular blood flow originates from the intracranial carotid arteries, and these therefore experience higher pulse pressures when compared to the vertebrobasilar arteries [31]. All of these factors are known to affect the development of arteriosclerosis and may account for the observed differences in density between ICAC and VBAC [32].

Previous investigations into calcification density in general are scarce and have focused almost exclusively on CAC. The Framingham Heart Study previously found CAC density to correlate with CAC volume to a similar extent as IAC density correlated with IAC volume in our comparably designed study, reporting a Spearman’s ρ of 0.75 [33]. The MESA Study previously reported a lower correlation between CAC density and CAC volume with a Spearman’s ρ of 0.56, suggesting density to be a different entity to volume, which holds implications for clinical scoring rules based on Agatston scores that incorporate both entities [7]. The moderate correlation of 0.67 in our results agrees with the suggestion that density is a separate entity from volume, with possibly different clinical characteristics and consequences.

Given the only moderate correlations between density and volume of IAC in this study, we explored potential differences between determinants of these two metrics. Almost all determinants of IAC density in our study exhibited similar effects when compared to determinants of IAC volume. Higher non-HDL cholesterol and smoking were observed to drive higher calcification densities, but not volume. Conversely, a history of CVA was found to drive higher calcification volumes, but not densities. All of these findings are mostly in line with what is known about determinants of CAC, indicating that the association between IAC volume and density resembles that of CAC volume and density [34].

Finally, we assessed differences in density between the two ICAC subtypes. The current visual scoring system for distinguishing these subtypes shows moderate reliability (intraclass correlation coefficients 0.64–0.82 [20]), indicating that there is some room for improvement in this system. The system may particularly struggle in borderline cases with scores near the threshold of ≥ 7, specifically in the range of 5 to 8, which encompasses the majority of our identified ICACs. In these instances, density is a readily quantifiable and objective marker, and our findings suggest that density can improve subtype classification where visual scoring alone is insufficient, particularly within this ambiguous scoring range. This improved discrimination holds implications for both research and clinical practice. In population-based studies, more accurate subtype identification reduces misclassification bias and enables more precise estimation of subtype-specific risks of cerebrovascular disease. This would particularly enhance studies on intracranial atherosclerotic disease, where poorer stroke treatment outcomes are associated more with intimal rather than medial calcifications [28]. For radiologists, incorporating density into routine assessment may facilitate clearer diagnoses of the type of intracranial arteriosclerosis on CT images, particularly in borderline cases. Subsequently, clinicians are enhanced in their assessment of each patient’s individual risk profile and their ability to communicate this risk assessment. However, these implications were not the subject of our investigation, given that we solely focused on determining the etiology of intracranial arteriosclerosis, and we therefore decided not to incorporate outcome data within this study. Future work combining histopathological samples with CT imaging is warranted to confirm the role of density in this context, which would in turn pave the way for studies that may investigate whether this improved distinction also leads to better risk management strategies, such as subtype-specific treatments or screening processes, and whether this improves patient outcomes.

### Strengths and limitations

The strengths of this study include its population-based design, which minimizes selection bias in the quantification of IAC metrics, thereby offering an advantage over patient-based studies. Furthermore, we employed a previously validated and reliable method to measure ICAC and VBAC, and our assessment of IAC density builds on this method as an extension. Though we did not validate this extended method directly, a recent report from our group demonstrated a strong agreement between mean density and volumetric assessments of carotid bifurcation calcification in a random sample of 100 participants, underscoring the reliability of our arteriosclerosis density measurements [35].

Several limitations require further discussion. While the imaging field-of-view and scanner settings were optimized to enable rating of IAC, only two CT systems were used for image acquisition, and we do not know how generalizable these findings are to CT systems of other vendors, nor to modern CT systems that offer higher resolution imaging. Moreover, the non-contrast CT images utilized in this study have a slice thickness of three millimeters, which may systematically bias results where smaller calcifications between slices are overlooked. This may have led to misclassification bias, particularly in early-stage arteriosclerotic disease, potentially leading to higher IAC volumes and densities. Nevertheless, non-contrast CT allows for the use of a lower minimum HU threshold for IAC detection compared to contrast-enhanced CT with a smaller slice thickness, enabling us to additionally assess lower-density calcifications. However, even non-contrast CT necessitates a bottom threshold of 130 HU to define IAC, and microcalcifications with a density lower than 130 HU will have been missed with this method, further limiting the generalizability of our findings to early-stage calcifications. Another limitation concerns the generalizability of this study to populations of different ethnic backgrounds. This study was conducted in an almost entirely white population. Given that the burden of CAC is known to vary significantly among ethnicities, with elevated burdens particularly among Asian and Hispanic populations compared to white populations [7], the same variability may hold for IAC density. IAC density may particularly vary in other ethnicities due to differences in cardiovascular determinants, such as increased incidence of hypertension, diabetes, dyslipidemia and cardiovascular disease, in addition to genetic and lifestyle differences associated with arteriosclerotic progression [36]. Furthermore, the CT technology applied in the present study may not be readily available in developing countries, further limiting the generalizability of our findings. Finally, in 89 participants, the ICAC subtype was visually scored in an artery where no ICAC volume could be measured. These participants were excluded from our analyses on the role of ICAC density in distinguishing the ICAC subtype, which may have led to more conservative estimates of this role.

To our knowledge, this study is the first to describe associations with cardiovascular risk factors and IAC density. This study, therefore, paves the way for further longitudinal research to determine whether IAC density plays a role in risk stratification for CVAs, akin to its role in CAC and myocardial infarction. Such a study holds great potential in informing strategies for the primary and secondary prevention of cerebrovascular diseases.

In conclusion, we identified distinct patterns of calcification density among different vessel beds and types of intracranial arteriosclerosis among older adults. Though density correlated only moderately with volume, the determinants of calcification density were similar to those of calcification volume, with notable differences for the effects of non-HDL cholesterol and smoking on increasing IAC density but not volume. These results suggest slightly different pathophysiologies underlying IAC density and volume, with each offering specific targets for prevention strategies.

## Supplementary information

ELECTRONIC SUPPLEMENTARY MATERIAL

## Abbreviations

CAC: Coronary artery calcification
CHD: Coronary heart disease
CI: Confidence interval
CVA: Cerebrovascular accident
HDL: High-density lipoprotein
IAC: Intracranial artery calcification
ICAC: Intracranial carotid artery calcification
IQR: Interquartile range
SD: Standard deviation
TIA: Transient ischemic attack
VBAC: Vertebrobasilar artery calcification
