# Supplementary material for: CT-derived density of intracranial arteriosclerosis: a population-based cohort study
Source: Eur Radiol. 2026 Jan 15;36(6):4862–72. doi: 10.1007/s00330-025-12180-1 (PMC13212371; doi:10.1007/s00330-025-12180-1)
Supplement: Supplementary file 1 — ELECTRONIC SUPPLEMENTARY MATERIAL [file 330_2025_12180_MOESM1_ESM.pdf]

CT-derived density of intracranial arteriosclerosis: a population-based cohort study

ELECTRONIC SUPPLEMENTARY MATERIAL

Supplementary Material

A. Elaborate description of the methodological framework behind measuring intracranial arteriosclerosis

First, axial CT slices were scrutinized using ImageJ (v. 1.46r, Rasband W.; National Institutes of Health, Maryland, USA) for the presence of arterial calcification among the cerebropetal arteries. This assessment encompassed arterial segments from the start of the petrous carotid canal and the foramen magnum, extending up to the top of the sella turcica. For intracranial carotid artery calcification (ICAC), the validated "Kockelkoren" scoring rule[1; 2] has previously been applied to visually determine the subtype of ICAC per artery.[3] This method uses visual identification of morphological characteristics using a composite score, with specific weighting for calcification circularity, thickness and continuity. Through this method, two subtypes are defined based on a score of < 7 points, indicating intimal calcifications (small, thick, irregular) often recognized in intracranial atherosclerotic disease[4], or ≥7 points indicating calcification of the internal elastic lamina (elongated, circular, thin) commonly termed medial calcification.[2; 3] The subtype of vertebrobasilar artery calcification (VBAC) was not determined, as this method is currently not yet validated for application to VBAC.

Next, arterial hyperdensities were manually delineated with a region of interest per artery, on each consecutive axial CT-slice where discernible. A volume of arterial calcification per artery was then automatically calculated using a custom-made plug-in

for ImageJ. This plug-in multiplies the number of voxels within the region of interest, possessing a density higher than the 130 Hounsfield Units (HUs)[5], by voxel dimension and slice increment. ICAC presence was determined by any volume of calcification exceeding 0 mm^3 among the intracranial carotid arteries. Similarly, VBAC presence was identified by any volume exceeding 0 mm^3 among the vertebral and basilar arteries. Total ICAC and VBAC volumes were computed by summing the corresponding arterial calcification volumes per arterial territory. Subsequently, IAC presence was defined by any volume of ICAC or VBAC exceeding 0 mm^3, and IAC volume was derived by summing ICAC and VBAC volumes.

References (also mentioned in main manuscript)

1. Vos A, Kockelkoren R, de Vis JB et al (2018) Risk factors for atherosclerotic and medial arterial calcification of the intracranial internal carotid artery. Atherosclerosis 276:44-49
2. Kockelkoren R, Vos A, Van Hecke W et al (2017) Computed Tomographic Distinction of Intimal and Medial Calcification in the Intracranial Internal Carotid Artery. PLoS One 12:e0168360
3. Van den Beukel TC, Van der Toorn JE, Vernooij MW et al (2022) Morphological Subtypes of Intracranial Internal Carotid Artery Arteriosclerosis and the Risk of Stroke. Stroke 53:1339-1347
4. Mazighi M, Labreuche J, Gongora-Rivera F, Duyckaerts C, Hauw JJ, Amarenco P (2008) Autopsy prevalence of intracranial atherosclerosis in patients with fatal stroke. Stroke 39:1142-1147
5. Agatston AS, Janowitz WR, Hildner FJ, Zusmer NR, Viamonte M, Jr., Detrano R (1990) Quantification of coronary artery calcium using ultrafast computed tomography. J Am Coll Cardiol 15:827-832
